# Factors associated with first- versus second-generation long-acting antipsychotics prescribed under ordinary clinical practice in Italy

**DOI:** 10.1371/journal.pone.0201371

**Published:** 2018-08-02

**Authors:** Giovanni Ostuzzi, Maria Angela Mazzi, Samira Terlizzi, Federico Bertolini, Andrea Aguglia, Francesco Bartoli, Paola Bortolaso, Camilla Callegari, Mariarita Caroleo, Giuseppe Carrà, Mariangela Corbo, Armando D’Agostino, Chiara Gastaldon, Claudio Lucii, Fabio Magliocco, Giovanni Martinotti, Michela Nosé, Edoardo Giuseppe Ostinelli, Davide Papola, Marco Piero Piccinelli, Alberto Piccoli, Marianna Purgato, Tommaso Tabacchi, Giulia Turrini, Mirella Ruggeri, Corrado Barbui, the STAR Network Investigators

**Affiliations:** 1 WHO Collaborating Centre for Research and Training in Mental Health and Service Evaluation, Department of Neuroscience, Biomedicine and Movement Sciences, Section of Psychiatry, University of Verona, Verona, Italy; 2 Department of Neuroscience, Rehabilitation, Ophthalmology, Genetics, Maternal and Child Health, Section of Psychiatry, IRCCS "Policlinico San Martino" Hospital, University of Genoa, Genoa, Italy; 3 "Rita Levi Montalcini" Department of Neuroscience, University of Turin, Turin, Italy; 4 Department of Medicine and Surgery, University of Milano Bicocca, Monza, Italy; 5 Università degli Studi dell'Insubria, Dipartimento di Salute Mentale e Dipendenze-ASST Settelaghi Varese, Varese, Italy; 6 Department of Health Sciences, Psychiatric Unit, University Magna Græcia of Catanzaro, Catanzaro, Italy; 7 Division of Psychiatry, University College of London, London, UK; 8 Department of Neuroscience, Imaging and Clinical Sciences, University "G. d'Annunzio", Chieti, Italy; 9 Department of Health Sciences, Università degli Studi di Milano, Milan, Italy; 10 Department of Mental Health, San Paolo Hospital, Milan, Italy; 11 Mental Health Department, USL Toscana sudest-Siena, Siena, Italy; Universita Cattolica del Sacro Cuore Sede di Roma, ITALY

## Abstract

**Background:**

For many years, long-acting intramuscular (LAI) antipsychotics have been prescribed predominantly to chronic and severe patients, as a last resort when other treatments failed. Recently, a broader and earlier use of LAIs, particularly second-generation LAIs, has been emphasized. To date, few studies attempted to frame how this change in prescribing took place in real-world practice. Therefore, this study aimed to describe the clinical features of patients prescribed with LAIs, and to explore possible prescribing differences between first- and second-generations LAIs under ordinary clinical practice in Italy.

**Methods:**

The STAR Network “Depot” Study is an observational, longitudinal, multicenter study involving 35 centers in Italy. In the cross-sectional phase, patients prescribed with LAIs were consecutively recruited and assessed over a period of 12 months. Descriptive statistics and multivariable logistic regression analyses were employed.

**Results:**

Of the 451 recruited patients, 61% were males. The level of social and working functioning was heterogeneous, as was the severity of disease. Seventy-two per cent of the patients had a diagnosis of the schizophrenia spectrum. Seventy per cent were prescribed with second-generation antipsychotic (SGA) LAIs (mostly paliperidone, aripiprazole and risperidone). Compared to first-generation antipsychotic (FGA) LAIs, patients prescribed with SGA LAIs were more often younger; employed; with a diagnosis of the schizophrenia spectrum or bipolar disorder; with higher levels of affective symptoms; with fewer LAI prescriptions in the past.

**Discussion:**

LAIs’ prescribing practices appear to be more flexible as compared to the past, although this change is mostly restricted to SGA LAIs.

## Introduction

The problem of non-adherence to medications is a major concern in people with psychotic disorders [1]. Antipsychotic discontinuation, is associated with poorer outcomes, including recurrent relapses and hospitalizations, higher risk of suicide, and earlier social and functional disability [2–4]. This is particularly relevant in early phases of disease, considering that discontinuation is particularly frequent within the first years from the first episode of psychosis (with estimated rates ranging from about 40% to 70%) [5,6], and most of these patients (from about 40% to 60% in most studies) are likely to relapse in the following few years [7,8]. Long-acting intramuscular (LAI) antipsychotics’ efficacy in preventing relapses and treatment discontinuation is widely recognized [6,9–11]. Further, LAIs allow a complete tracking of medication intake, promote the regular monitoring of clinical conditions, and lower the risk of self-medication and harmful drug use. Also relevant disadvantages have been highlighted, such as pain on the injection site, lack of flexibility in dose adjustments, and the impossibility of rapid suspension in case of adverse events [12,13].

From their introduction, LAIs have been generally considered as a last-resort for chronic patients with frequent relapses, low insight of disease, poor adherence to treatments, aggressiveness and behavioral issues [12,14–16]. However, in the last decade, various factors contributed to change this scenario. First, in addition to risperidone (marketed since 2001), other second-generation antipsychotic (SGA) LAIs became available: olanzapine pamoate (since 2009), paliperidone palmitate 1-month (since 2009), aripiprazole long-acting (since 2010), and paliperidone palmitate 3-months (since 2015) [12,17]. Second, in the same years, growing evidence emerged in support of the use of antipsychotics (SGAs in particular) not only for schizophrenia, but also for affective disorders [18,19]. Third, as the huge impact of non-adherence on the course of disease became more evident, emphasis has been put on the need to address this issue from the earliest phases of the disease [20,21]. Fourth, studies exploring the subjective experience of patients suggest that the practicality of LAIs can notably ease patients’ daily routine, possibly improving attitudes toward medications [22–28]. These findings contributed to progressively overcome the culturally rooted view of LAIs as coercive and stigmatizing medications [12,14,29,30].

On these grounds, many researchers advocated for an earlier and broader use of LAIs [20,21,31–34], and this viewpoint has been accounted by the most influential clinical guidelines [35–38].

Despite the backdrop, few studies attempted to describe how this novel paradigm affected prescribing practices. Available epidemiological data are limited to schizophrenia patients [39–43] and highly selected populations [40,44,45]. In order to fill the gap in the research literature, the cross-sectional phase of the STAR Network “Depot” Study aimed at evaluating how the claimed cultural change in LAIs prescription may have affected real-world practices, by (a) describing the main socio-demographic and clinical features (including symptom profiles, adherence and attitude towards treatments) of a large, representative, unselected population of patients starting a LAI medication, and (b) exploring possible predictors of the class of LAI prescribed.

## Materials and methods

### Study design

The STAR Network “Depot” Study is an observational, longitudinal, multicenter study. Patients prescribed with any LAI antipsychotic were consecutively recruited over a period of 12 months, and assessed after 6 and 12 months. The present paper is focused on baseline data from the recruitment phase.

Participating centers are part of the STAR Network (*Servizi Territoriali Associati per la Ricerca—*Community Services Associated for Research), a consortium of clinicians and researchers working in Community Psychiatric Services across Italy. The main aim of this group is to gather original data from real-world clinical practice and provide new pragmatic insights for clinicians [46–48]. The STAR Network “Depot” Study was conducted independently from industry funding or support. The study protocol was approved by the Local Ethics Committee of the Ethics Committee of the *Azienda Ospedaliera Universitaria Integrata* of Verona (*Comitato Etico per la Sperimentazione Clinica (CESC)* of the Provinces of Verona and Rovigo, protocol n. 57622 of the 09/12/2015), and was made publicly available at the Open Science Framework (OSF) online repository (https://osf.io/wt8kx/). The present study was drawn up following the ‘STrengthening the Reporting of OBservational studies in Epidemiology’ (STROBE) Statement items [49].

### Participants

Patients were eligible for the study if they were (a) at least 18 years old; (b) willing to sign the informed consent; and (c) beginning a LAI medication, provided that they did not receive any other LAI in the previous three months. The simultaneous taking of other medications, including oral antipsychotics, was not an exclusion criterion, nor was the setting of recruitment, which might include hospital psychiatric wards, daytime community and residential facilities.

### Measures

In order to collect socio-demographic and clinical data, the following measures were administered at the baseline evaluation:

▪ Recruitment form, which included socio-demographic information, clinical and pharmacological information, and characteristics of the clinician who prescribed the LAI;
▪ The clinician-rated Brief Psychiatric Rating Scale (BPRS) [50], validated in Italian language [51], which assesses overall psychiatric symptoms. The overall level of symptomatology should be considered mild, moderate and severe for scores ranging respectively from 31 to 40, 41 to 52 and higher than 52 [52]. According to Shafer and colleagues [53], five subscales were measured, namely affect, positive symptoms, negative symptoms, resistance, and activation;
▪ The self-administered Drug Attitude Inventory 10 items (DAI-10) [54], validated in Italian language [55], which measures attitudes toward medications. The scores range between -10 and 10, with higher scores indicating a better overall attitude toward medications;
▪ The clinician-rated Kemp’s 7-point scale [56], compiled by the clinician, which assesses overall adherence to treatments. The scores range from 1 to 7, with higher scores indicating equally higher levels of adherence. Scores of 5 and above indicate an overall good acceptance of medications.

### Data management

All patients received detailed information on the nature of the study and the use of data on an anonymised format, and were asked to sign an informed consent. The recruiter was required to examine the mental capacity of the patients, by assessing his/her capacity of understand both in a strict sense (i.e. comprehension of the language) and in a broad sense (i.e. the context, the role of the examiner, the content of the research project, etc.), and to communicate his/her decision, in order to determine whether he/she was capable to freely provide written consent. Recruitment data were periodically forwarded from each recruiting center to the Verona center, which had the role of coordination and scientific secretariat. Data were archived and entered into a computerized database. Their correctness and consistency was ensured by the double-entry technique and by a set of electronic and manual edit checks. Patient data were recorded anonymously. A unique number both in the recruitment and follow-up forms and in the database identified patients. Total confidentiality of data was guaranteed throughout the entire course of the study, in accordance with the Declaration of Helsinki [57].

### Statistical analysis

All statistical analyses were performed with STATA 13.0 [58]. Descriptive statistics were used for information on main epidemiological characteristics of the recruited population. Continuous variables were expressed as means and standard deviations, while categorical variables were expressed as percentages. In order to describe possible associations between clinical and socio-demographic characteristics and the class of LAI prescribed, both simple and multivariable logistic regression analyses were performed. A bivariate analysis employing the class of LAI as the dependent variable (0 = first-generation antipsychotic (FGA) LAIs; 1 = SGA LAIs) was applied to a number of variables of clinical relevance. For the aims of the logistic model, we employed the following dichotomous and categorical data: Italians versus non-Italians; poor autonomy level (lives with relatives or in a residential house) versus good autonomy level (lives alone or with partner and/or children); diploma/University degree versus other; employed versus unemployed; schizophrenia spectrum versus bipolar disorder versus other diagnosis; academic versus non-academic centers; Northern Italy versus Central and Southern Italy. We also calculated the ratio between the prescribed daily dose (PDD) in the previous 12 months and the defined daily dose (DDD) [59]. All variables for which a statistically significant association emerged in the bivariate analysis were included as independent variables in a multivariable model. Subsequently, we produced a final multivariable model, including only the independent variables for which a statistically significant association emerged from the previous multivariable model. In order to evaluate the adequacy of the models some indices of goodness of fit were performed. Specifically, the Hosmer-Lemeshow test, based on the comparison between predicted and observed data by using deciles as stratification size, assesses whether the model is well calibrated; McKelvey & Zavoina’s pseudo R^2^, for its interpretation (as percentage of explained variance) closed to the “traditional” way [60]. Finally, Bayesian Information Criterion (BIC) was used in the comparison between intermediate and final multivariable models, to determine whether the exclusion of some explanatory variables decreased the explanatory power of the last model. Regression analyses were based on robust estimator of variance (*cluster* option of the STATA *vce* command) to account for the multicenter observational design [61].

## Results

### Participating centers

Thirty-five Italian Community Psychiatric Centers took part in the study. Recruiting centers included mostly non-academic mental health trusts (73.4%), however, the number of patients recruited was equally distributed between academic and non-academic centers (54.5% vs. 45.4%, respectively). The majority of centers (73.4%) were located in Northern Italy, however the number of patients recruited in these centers was only slightly higher than the number recruited in Central and Southern Italy (59.4% vs. 40.6%, respectively).

### Socio-demographic and clinical features

We recruited and included in the analysis 451 patients. All included participants provided an informed, written consent. Sixty-one per cent of patients were males, with a mean age of 42. Most patients lived with relatives (51%). Forty per cent had a diploma and 10% a University degree. About 22% were employed at the time of recruitment. Other socio-demographic details are reported in Table 1.

**Table 1:** Socio-demographic features.

**Variables**	**All LAIs, n = 451**
Age, mean (sd)	41.8 (13.4)
Age categories, n (%)	
18–30	111 (24.6)
31–45	161 (35.7)
46–60	144 (31.9)
>61	35 (7.8)
Female, n (%)	177 (39.2)
Italian, n (%)	390 (88.2)
Housing conditions, n (%)	
Alone	100 (22.2)
With partner and/or children	95 (21.1)
With other relatives	229 (50.8)
Any residential home	27 (6)
Marital status, n (%)	
Non-conjugated	383 (85.1)
Conjugated	67 (14.9)
Educational level, n (%)	
Illiterate/no title	7 (1.6)
Primary school	27 (6.1)
Secondary school	189 (42.5)
Diploma	178 (40)
University degree	44 (9.9)
Work, n (%)	
Employed	100 (22.2)
Unemployed	221 (49)
Student	15 (3.3)
Retired	68 (15.1)
Housewife/other	47 (10.4)

LAI = long-acting injection; n = number of patients; sd = standard deviation

**Fig 1:**
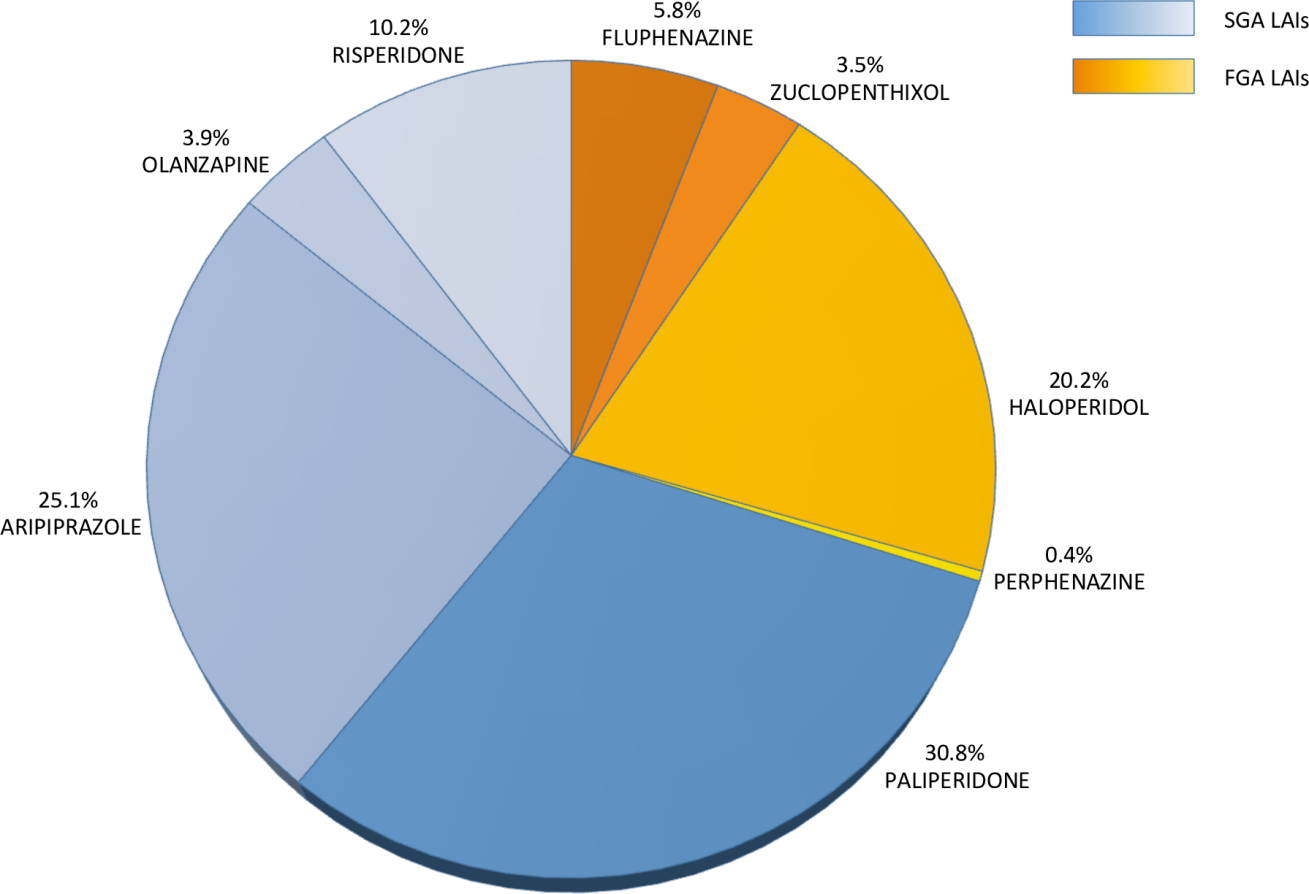
Frequency of LAIs prescribed. Legend: SGA = second-generation antipsychotic; FGA = first-generation antipsychotics; LAI = long-acting injection.

Fifty-six per cent suffered from schizophrenia, 16.5% from schizoaffective disorder, 18% from bipolar disorder, 6% from personality disorders, and the remaining 3.5% from other conditions, including obsessive-compulsive disorder, substance use disorders, mental retardation, mental organic disorders, and dementia. We found high rates of alcohol and/or substance abuse (27%) and of physical comorbidity (28%). According to the mean BPRS score, the level of psychiatric symptoms was negligible for 12% of patients, mild for 19%, moderate for 26%, and severe for 42%. Fifty-nine per cent of patients showed a positive attitude toward medications according to the mean DAI-10 score, and 61% showed a good acceptance of medications according to the mean Kemp’s 7-point score. Ninety-two per cent of patients were taking at least one oral psychotropic medication before introducing the LAI. The mean ratio between the prescribed daily dose (PDD) and the defined daily dose (DDD) of all psychotropic drugs taken in the previous year was 1.8 (sd 2), indicating that the cumulative dose of medications was almost doubled with respect to the average maintenance dose. The majority of patients were prescribed paliperidone palmitate (31%), aripiprazole LAI (25%), haloperidol decanoate (20%) and risperidone LAI (10%) (Fig 1). Other clinical and pharmacological details are reported in Table 2.

**Table 2:** Clinical features.

**Variables**	**All LAIs, n = 451**
Diagnosis, n (%)	
Schizophrenia	251 (55.9)
Schizoaffective disorder	74 (16.5)
Substance-related psychosis	2 (0.4)
Bipolar disorder	81 (18)
Obsessive-compulsive disorder	4 (0.9)
Personality disorder	27 (6)
Mental retardation	4 (0.9)
Mental organic disorder	4 (0.9)
Dementia	2 (0.4)
Time from disease onset, mean years (sd)	11.9 (10)
Alcohol use, n (%)	65 (14.4)
Any substance use, n (%)	90 (19.9)
Polysubstance use, n (%)	14 (3.1)
Substances, n (%)	
Cannabis	69 (76.7)
Cocaine	13 (14.4)
Other	8 (8.9)
At least one medical comorbidity, n (%)	127 (28.2)
Medical comorbidity, n (%)	
Infective disease	8 (6.3)
Endocrine/metabolic disease	48 (37.8)
Cardiovascular disease	23 (18.1)
Neurologic disease	10 (7.9)
Gastrointestinal disease	11 (8.7)
Other	27 (21.2)
BPRS, mean (sd)	49 (14.7)
BPRS positive symptoms, mean (sd)	12.1 (5.4)
BPRS negative symptoms, mean (sd)	7.8 (3.7)
BPRS affective symptoms, mean (sd)	10.5 (4.3)
BPRS resistance, mean (sd)	9.4 (4.5)
BPRS activation, mean (sd)	7.6 (3.3)
DAI-10, mean (sd)	2 (5.3)
Kemp’s 7-point scale, mean (sd)	4.8 (1.4)
At least one hospitalization in the last year, n (%)	270 (59.9)
At least one compulsory hospitalization, n (%)	89 (19.7)
Length of hospitalizations, mean days (sd)	22.7 (19.5)
Cumulative dose of psychotropic drugs taken in the last year: PDD/DDD, mean (sd)	1.8 (2)
LAIs PDD/DDD, mean (sd)	1.3 (1.2)
Number of previous LAIs, n (%)	
0	316 (70.1)
1	103 (22.8)
2 or more	32 (7.1)

n = number of patients; sd = standard deviation; BPRS = brief psychiatry rating scale; DAI = drug attitude inventory; PDD = prescribed daily dose; DDD = defined daily dose

### Comparison between classes of antipsychotic

Table 3 reports the comparison between FGA LAIs and SGA LAIs for a number of clinically relevant variables. Raw data of each group, the bivariate analysis, and the two subsequent multivariable analyses are reported. The bivariate analysis showed eleven independent variables to be significantly associated with a higher prescription of SGA LAIs, and the two subsequent multivariable models confirmed this significant association for the following variables: younger age (OR 0.97; 95% CI 0.95 to 0.98); being employed (OR 2.01; 95% CI 1.14 to 3.56); having a higher score on the BPRS affective subscale (OR 1.09; 95% CI 1.04 to 1.14); and having a lower number of LAIs prescribed in the past (OR 0.69; 95% CI 0.52 to 0.93). In addition, having a diagnosis other than schizophrenia spectrum and bipolar disorder reduced the probability of receiving SGA LAIs (OR 0.28; 95% CI 0.13 to 0.60).

**Table 3:** Bivariate and multivariable comparison between FGAs and SGAs.

Variables	FGAs LAIs,n = 135	SGAs LAIs,n = 316	SGAs vs. FGAs		
			unadjusted OR[95% CI]	adjusted OR*[95% CI]	adjusted OR**[95% CI]
Age, mean (sd)	45.9 (13.2)	40.1 (13.2)	**0.97 [0.95 to 0.98]**	**0.97 [0.95 to 0.99]**	**0.97 [0.95 to 0.98]**
Female, n (%)	60 (44.4)	117 (37.1)	0.73 [0.49 to 1.11]	-	-
Italian, n (%)	122 (91)	268 (87)	1.52 [0.77 to 2.99]	-	-
Lives alone or with partner/children, n (%)	69 (51.1)	126 (39.9)	**0.63 [0.42 to 0.95]**	0.85 [0.49 to 1.46]	-
Diploma or University degree, n (%)	58 (43.9)	164 (52.4)	1.40 [0.93 to 2.11]	-	-
Employed, n (%)	21 (15. 6)	79 (25)	**1.81 [1.06 to 3.07]**	**1.99 [1.02 to 3.90]**	**2.01 [1.14 to 3.56]**
Diagnosis, n (%)					
Schizophrenia spectrum	92 (68.1)	233 (74.2)	ref.	ref.	ref.
Bipolar disorder	21 (15.6)	60 (19.1)	1.13 [0.65 to 1.96]	1.11 [0.53 to 2.30]	1.09 [0.52 to 2.31]
Other	22 (16.3)	21 (6.7)	**0.38 [0.20 to 0.72]**	**0.30 [0.14 to 0.67]**	**0.28 [0.13 to 0.60]**
BPRS, mean (sd)	48.3 (13.1)	49.3 (15.4)	1.00 [0.99 to 1.02]	-	-
BPRS affective symptoms, mean (sd)	9.5 (3.9)	10.9 (4.4)	**1.08 [1.03 to 1.14]**	**1.10 [1.05 to 1.15]**	**1.09 [1.04 to 1.14]**
BPRS positive symptoms, mean (sd)	11.9 (4.8)	12.2 (5.6)	1.01 [0.97 to 1.05]	-	-
BPRS negative symptoms, mean (sd)	7.4 (3.5)	7.9 (3.7)	1.04 [0.98 to 1.10]	-	-
BPRS resistance, mean (sd)	10.1 (4.4)	9.1 (4.5)	**0.95 [0.91 to 0.99]**	0.96 [0.90 to 1.03]	-
BPRS activation, mean (sd)	7.6 (3.1)	7.6 (3.5)	1.00 [0.94 to 1.06]	-	-
DAI-10, mean (sd)	1.1 (5.5)	2.4 (5.3)	**1.05 [1.01 to 1.09]**	1.02 [0.96 to 1.07]	-
Kemp’s 7-point scale, mean (sd)	4.5 (1.5)	4.9 (1.4)	**1.24 [1.08 to 1.44]**	1.02 [0.78 to 1.35]	-
N. of hospitalizations in the last year, mean (sd)	1 (1.1)	0.8 (1.1)	**0.82 [0.69 to 0.99]**	0.86 [0.66 to 1.11]	-
Length of hospitalizations (days), mean (sd)	14.3 (17.3)	13.4 (19.3)	1.00 [0.99 to 1.01]	-	-
At least one compulsory hospitalization, n (%)	33 (35.1)	56 (31.8)	0.86 [0.51 to 1.46]	-	-
Alcohol abuse, n (%)	21 (15.6)	44 (13.9)	0.88 [0.50 to 1.54]	-	-
Substance abuse, n (%)	27 (20)	63 (19.9)	1.00 [0.60 to 1.65]	-	-
At least one medical comorbidity, n (%)	48 (35.6)	79 (25.1)	**0.61 [0.39 to 0.94]**	0.82 [0.54 to 1.26]	-
Number of previous LAIs, mean (sd)	0.5 (0.7)	0.3 (0.6)	**0.67 [0.50 to 0.90]**	**0.73 [0.55 to 0.96]**	**0.69 [0.52 to 0.93]**
Number of psychotropic drugs in the last year, mean (sd)	1.5 (1.1)	1.3 (1)	0.88 [0.73 to 1.08]	-	-
Cumulative dose of psychotropic drugs taken in the last year: PDD/DDD, mean (sd)	1.6 (1.6)	1.88 (2.2)	1.08 [0.96 to 1.22]	-	-
University center, n (%)	77 (57)	169 (53.5)	0.86 [0.58 to 1.30]	-	-
South-center Italy, n (%)	51 (37.8)	132 (41.8)	1.18 [0.78 to 1.79]	-	-
Prescriber’s age, mean (sd)	46.7 (12.3)	45.6 (10.4)	0.99 [0.97 to 1.01]	-	-
**Goodness-of-fit**					
H-L Chi^2^ (p-value)				8.09 (0.43)	14.71 (0.06)
M&Z pseudo R^2^				0.18	0.16
BIC				566.712	540.875

* the intermediate multivariable model includes only variables for which a statistically significant association emerged in the bivariate analysis

** the final multivariable model includes only variables for which a statistically significant association emerged from the intermediate model

Bold characters indicate a p-value < 0.05.

The % reported in parenthesis refers to the ratio calculated respectively on all LAIs (first column), FGA LAIs (second column), SGA LAIs (third column)

SGA = second-generation antipsychotic; FGA = first-generation antipsychotic; n = number of patients; sd = standard deviation; OR = odds ration; CI = confidence interval; BPRS = brief psychiatry rating scale; DAI = drug attitude inventory; PDD = prescribed daily dose; DDD = defined daily dose; H-L Chi^2^ = Chi-squared value of the Hosmer-Lemeshow test; M&Z pseudo R^2^ = McKelvey-Zavoina pseudo R^2^ index; BIC = Bayesian information criterion

The goodness-of-fit statistics indicated a moderate relationship between the type of LAIs prescribed and the explanatory variables in both models: McKelvey and Zavoina Pseudo-R^2^ explained 18% and 16% of the variability. In calibration view, the models correctly classified 73% and 72% of the patients; the p-values associated with Hosmer–Lemeshow statistics were low even though not significant, confirming moderate agreements between predicted probabilities and observed data. Finally, the comparison between the BIC values showed a substantial difference (25.84), preferring the final model.

## Discussion

In general, present data confirm the expectation that a broader spectrum of individuals is currently prescribed LAIs as compared to the past. A wide range of socio-demographic and clinical characteristics emerged. A relevant part of the cohort met the features typically described in older studies (before the broad availability of most SGA LAIs), as patients were mostly males in their middle adulthood, with low educational level, no employment, a long-standing diagnosis of schizophrenia, and moderate-to-severe level of psychiatric symptoms [15,62–65]. At the same time, relatively high functioning levels emerged in a surprisingly large part of the population, considering that about 43% of patients lived alone or with the partner and/or children, more than one out of five patients were employed, and half of the patients had a diploma or a University degree. Similar considerations apply to clinical features, considering that, beside a large number of patients with chronic conditions and severe symptomatology, also patients with mild-to-moderate levels of symptomatology were well represented. Further, data showed a relatively short course of disease (lower than 5 years) in about 36% of patients, and an overall good attitude towards medications as perceived by both patients and clinicians. Interestingly, a variety of diagnoses emerged. High rates of bipolar disorder were expected, considering the recently broadened use of antipsychotics for affective disorders [18,19]. In about 6% of patients, the LAI was probably prescribed to manage severe behavioral symptoms arising from personality disorders or underlying somatic conditions (such as mental retardation or dementia), although the use of antipsychotics in these cases is at least controversial [66,67].

Highly selected populations from previous studies can be barely compared with the present study, which employed a pragmatic, naturalistic approach, aimed at minimizing patients’ selection and reflect real-world practice as closely as possible.

The use of LAIs on a broader number of clinical conditions may raise regulatory issues, considering that licensed indications of SGA LAIs are limited only to patients with schizophrenia in a maintenance phase with oral antipsychotics. Therefore, SGA LAIs were prescribed off-label to all patients without a diagnosis of schizophrenia (almost one out of five patients). On the contrary, indications of FGA LAIs are narrower, often referring to symptom domains rather than specific diagnosis, and may therefore be prescribed to patients with several different diagnoses. The common off-label prescription of LAIs confirms the already well-known trend of oral antipsychotics [68].

Representativeness of the cohort seems warranted also in terms of high prevalence of comorbid physical conditions [69,70] (about one out of four patients had at least one comorbidity, mostly endocrine/metabolic or cardiovascular) and of “dual diagnosis”, considering that one out of four used alcohol or substances [71–73].

In most cases LAIs were prescribed after severe disease relapse, considering the large number of patients hospitalized in the previous year, the high rate of compulsory admissions, and the long mean length of stay. This may suggest that, despite the recommendation of offering LAIs from the early phases of disease [37,38], in many cases these formulations are still chosen after failures with other treatments.

More than two out of three patients were prescribed with SGA LAIs. The most commonly prescribed medications were paliperidone palmitate (31%), aripiprazole LAI (25%) and haloperidol decanoate (20%). These results are in line with data from some of the previous studies [42,74,75], while, in others, prescription rates appeared to be extremely heterogeneous [40,41,44]. This is likely to be associated with a number of factors influencing local prescribing patterns and with the different recruitment periods across studies (and therefore different availability of SGA LAIs). The use of aripiprazole LAI was surprisingly high compared to other recent studies [40–42,75]. The advantages of this medication have been repeatedly stressed: it is relatively safe in terms of motor, metabolic and endocrine side effects (it does not usually alter prolactin levels), and it proved to be comparable to other SGAs in terms of efficacy for the treatment of schizophrenia [76,77]. Further, robust results from a recent meta-analysis showed a better overall acceptability of aripiprazole LAI as compared to the oral counterpart, although the interpretation of this data is still unclear [78]. On the other hand, paliperidone substantially equals olanzapine and risperidone in terms of metabolic effects and prolactin level increase [76]. Its choice over olanzapine and risperidone is likely to be related to its enhanced practicality compared to the biweekly administration of risperidone LAI, and the complex regulatory requirements of olanzapine pamoate. Haloperidol decanoate, besides its possible disadvantages (e.g. motor symptoms, QTc prolongation, locally irritant preparations), remains a widely used medication in clinical practice, possibly because of its relatively safe metabolic profile [76], its low cost, and the flexibility of the LAI in terms of doses and frequency, as compared to other LAIs (including SGAs).

The multivariable logistic model comparing FGA LAIs and SGA LAIs showed that the latter were prescribed significantly more often to younger, employed individuals, with a diagnosis of schizophrenia or bipolar disorder, with higher levels of affective symptoms, and without a previous history of LAI prescription. This profile resembles closely the one pictured by those claiming a cultural change in the clinical use of LAIs. This trend is similar to what emerged from previous studies, although in many cases only FGA LAIs and risperidone LAI were compared [79–81], confounders were not considered [40,42,80], and social and clinical variables possibly associated with the class of LAI were not analyzed. Notably, no significant differences emerged between patients prescribed with FGA LAIs and SGA LAIs in terms of overall psychiatric symptoms, adherence and attitudes towards medications, both as perceived by psychiatrists (Kemp’s 7-point score) and by patients (DAI-10 score). To our knowledge, the study by Singh and colleagues [81] is currently the only available one employing the DAI (in this case, the version with 30 items), and it reached similar conclusions, although only FGA LAIs and LAI risperidone were compared.

As expected according to current research [82–84], SGAs were preferred when targeting affective symptoms. This may also reflect the common idea of FGAs as medications associated with apathy, lack of initiative, anhedonia, indifference, blunted affect (the so-called neuroleptic-induced deficit syndrome) [85,86].

This study has also limitations. First, our cross-sectional design cannot detect a causal association between variables, and all statistical associations discussed should be regarded as merely exploratory. Second, the use of simple rating scales (in accordance to the pragmatic attitude of the study) might have affected the precision in measuring variables, such as psychiatric symptoms and patients’ attitudes toward medications. Third, characteristics of recruiting centers were heterogeneous in terms of settings (e.g. community services, hospital wards, rehabilitation facilities), and each site contributed to the recruitment to a different extent. In addition, various local factors may have strongly influenced prescribing attitudes of each center (e.g. local guidelines and long-standing habits, availability of medications). This, along with our wide inclusion criteria, led to heterogeneity in the recruited population. This reflects the complexity of real-world clinical settings, bolstering the external validity of results, but may at the same time affect their internal validity [87]. To address this limitation, we employed statistical techniques accounting for variability between centers.

## Conclusions

This study showed a notable change in LAIs prescribing patterns, as compared with previous epidemiological surveys. The advocated cultural change in the use of LAIs is currently under way in Italian Community Psychiatric Services, as showed by more flexible and heterogeneous prescribing patterns, addressing a wider range of clinical conditions and functioning levels. This change appears to be mostly confined to SGA LAIs, while prescribing patterns of FGA LAIs are practically unchanged as compared to the past, as they are mostly reserved to older patients, with lower functioning levels, previous use of LAIs, and, commonly, behavioral issues.

Further research is needed to shed light on how LAIs can improve adherence, attitudes toward medications, and subjective wellbeing of patients in real-world practice. This will be the main goal of the follow-up phase of the STAR Network “Depot” Study.
